# Transcriptional Regulation and Its Misregulation in Human Diseases: Recent Progress

**DOI:** 10.3390/ijms26209941

**Published:** 2025-10-13

**Authors:** Alfredo Ciccodicola, Amelia Casamassimi, Monica Rienzo

**Affiliations:** 1 Institute of Genetics and Biophysics “Adriano Buzzati-Traverso”, CNR, Via P. Castellino 111, 80131 Naples, Italy; 2 Department of Science and Technology, University of Naples “Parthenope”, 80143 Naples, Italy; 3 Department of Precision Medicine, University of Campania “Luigi Vanvitelli”, Via L. De Crecchio, 80138 Naples, Italy; amelia.casamassimi@unicampania.it; 4 Department of Environmental, Biological, and Pharmaceutical Sciences and Technologies, University of Campania “Luigi Vanvitelli”, 81100 Caserta, Italy; monica.rienzo@unicampania.it

Transcriptional regulation is a central and finely orchestrated process through which cells control gene expression in a spatially and temporally coordinated manner. This regulation involves a dynamic interplay between transcription factors, cofactors, epigenetic modifiers, noncoding RNAs (ncRNAs), and chromatin architecture, allowing cells to respond precisely to developmental cues and environmental stimuli. As highlighted in the seminal review by Casamassimi et al. [1], the human transcriptome is shaped not only by the activity of RNA polymerase II (RNApol II) and transcription factor (TF) binding but also by the structural configuration of chromatin, nucleosome positioning, histone modifications, DNA methylation, and RNA-mediated mechanisms. These multilayered levels of control contribute to the establishment and maintenance of cellular identity and tissue specificity throughout life.

The complexity of this system enables precise control of gene expression, but it also renders it susceptible to disruption. Genetic mutations, epigenetic alterations, and aberrant signaling can all perturb transcriptional programs, contributing to the onset and progression of various human diseases. Notably, the development of high-throughput genomic and transcriptomic approaches has significantly advanced our understanding of transcriptional control and its misregulation. At the same time, the huge amount of online available datasets has allowed the improvement of bioinformatic tools to analyze the genomic and transcriptomic results [1]. Previously [2], we underscored how the misregulation of transcriptional networks is a unifying feature across numerous pathological conditions, from cancer and cardiovascular diseases to neurodegenerative and immune disorders. These insights stress the relevance of studying transcriptional mechanisms not only to understand disease etiology but also to identify new biomarkers and therapeutic targets.

This Special Issue brings together six interesting contributions—three original research articles and three reviews—that explore distinct facets of transcriptional regulation in human health and disease.

Importantly, all of the contributions included in this Special Issue offer novel perspectives on the molecular mechanisms underlying human diseases. These studies aim to uncover potential diagnostic biomarkers and therapeutic targets, with a strong emphasis on cancer and other complex disorders.

In the first research article, Utjés et al. [3] investigated the transcriptomic response of healthy breast tissue to short-term exposure to mifepristone—a selective progesterone receptor antagonist—in a randomized, double-blind, placebo-controlled trial involving premenopausal women. Using RNA sequencing (RNA-seq), the authors identified significant differential expression of genes related to extracellular matrix remodeling, several of which are known to be dysregulated in breast cancer development. Notably, their findings suggest that antiprogestins, such as mifepristone, may exert preventive effects on hormone-responsive breast tissues, providing a molecular rationale for their use in chemoprevention strategies. This study represents one of the few transcriptome-wide analyses of the in vivo effects of progesterone receptor modulation in human breast tissue, offering important insights into hormonal control mechanisms and their potential therapeutic exploitation.

In the second work, Delihas [4] presented a detailed evolutionary and functional analysis of a human-specific de novo open reading frame (ORF) located within the SMIM45 locus, accompanied by a linked transcriptional silencer element. Through comparative genomic analyses across multiple primate species and other mammals, the study demonstrates how this ORF originated from a noncoding region and acquired transcriptional activity specifically in the human lineage. Moreover, the author identified a cis-acting silencer sequence that may regulate expression of the ORF, suggesting an additional layer of transcriptional control. These findings provide compelling evidence for the emergence of novel regulatory and coding elements during evolution and highlight their possible roles in shaping human-specific traits or disease susceptibility. The study exemplifies how genome evolution can generate functionally relevant regulatory modules de novo, offering new perspectives on molecular evolution and transcriptomic diversity. 

The epigenetic mechanisms underlying cardiac cell identity, focusing on the Chd4/NuRD chromatin remodeling complex and its interaction with the transcription factor Znf219, are the subject of the study explored by El Abdellaoui-Soussi and colleagues [5]. Through transcriptomic profiling and loss-of-function experiments in mouse embryonic hearts and cardiomyocytes, the authors demonstrated that Znf219 is essential for the maintenance of cardiac-specific gene expression programs, particularly those related to sarcomeric structure and function, also suggesting that their misregulation may lead to the induction of arrhythmias, cardiac fibrosis and heart failure. Mechanistically, Znf219 was shown to cooperate with Chd4 to repress non-cardiac gene expression, thereby preserving cardiac lineage fidelity. Thus, this study highlights the crucial role of chromatin remodeling factors in safeguarding tissue-specific transcriptional landscapes and provides valuable insights into the regulatory networks that govern heart development and disease.

An important role in transcriptional regulation is also played by ncRNAs, including circular RNAs (circRNAs), which are emerging as key regulators of gene expression. These molecules can modulate transcriptional and post-transcriptional processes through various mechanisms, such as miRNA sponging, interaction with RNA-binding proteins, and modulation of TF activity. In this context, Long et al. [6] provided a comprehensive review of the current knowledge on circRNAs and their involvement in cardiovascular diseases (CVDs). CircRNAs, characterized by their covalently closed loop structures, exhibit high stability and tissue-specific expression patterns. The authors described the molecular mechanisms underlying circRNA biogenesis, such as back-splicing and exon skipping, and highlighted their regulatory roles in cardiac hypertrophy, fibrosis, myocardial infarction, and heart failure. Moreover, the review emphasized the diagnostic and therapeutic potential of circRNAs in CVDs, given their detectability in body fluids and disease-specific expression profiles. Finally, the authors discussed key challenges in the field, including the need for functional validation, mechanistic studies, and translational approaches to bring circRNA-based tools closer to clinical application.

In the second review, Gárate-Rascón et al. [7] focused on SLU7, a multifunctional gene expression regulator originally identified as a splicing factor and now recognized as a key integrator of multiple layers of transcriptional control. The authors discussed how SLU7 participates not only in pre-mRNA splicing, but also in processes such as epigenetic regulation, transcriptional activity, mRNA stability, protein ubiquitination, and subcellular localization. Particular attention was given to SLU7’s role in liver homeostasis, where it maintains chromatin integrity and prevents hepatocyte dedifferentiation. Furthermore, dysregulation of SLU7 expression or function has been implicated in hepatocellular carcinoma, colorectal cancer, and neurological disorders, underscoring its relevance in both physiological and pathological contexts. This review offers a broad and mechanistic perspective on how a single regulatory protein can influence gene expression at multiple checkpoints, making SLU7 a potential target for therapeutic modulation in various diseases.

In the third review, Kim and Shin [8] provided an in-depth analysis of Signal Transducer and Activator of Transcription 5 (STAT5), a transcription factor family that plays a central role in mediating cytokine and growth factor signaling. The authors summarized the structural features, activation mechanisms, and downstream targets of STAT5A and STAT5B, highlighting their involvement in critical biological processes such as immune cell development, hematopoiesis, metabolism, and cell proliferation. Emphasis was placed on how gain-of-function mutations, aberrant activation, or altered nuclear localization of STAT5 contribute to the pathogenesis of hematological malignancies, solid tumors, and immune-mediated diseases. The review also examined current pharmacological strategies aimed at inhibiting STAT5 signaling, including small molecule inhibitors and peptide mimetics, and discussed their potential therapeutic applications. Overall, this contribution offers a comprehensive overview of STAT5 as a molecular hub linking extracellular signals to transcriptional programs, and as a promising target for precision medicine approaches in cancer and immune disorders.

As our understanding of transcriptional regulation deepens, several emerging technologies are poised to revolutionize both the investigation and therapeutic modulation of gene expression. Among these, the application of CRISPR-based epigenome and transcriptome editing systems is particularly promising. Beyond conventional genome editing, CRISPR/dCas9-fused effectors allow for precise, locus-specific modulation of transcription—either by recruiting activators or repressors, or by altering chromatin states without introducing double-stranded breaks [9,10]. These approaches enable the functional interrogation of noncoding regulatory elements, enhancer–promoter interactions, and the therapeutic reactivation or silencing of disease-associated genes.

Simultaneously, the integration of artificial intelligence (AI) and machine learning (ML) into transcriptomic research is transforming our ability to analyze and interpret complex gene expression data. AI-driven models are now used to predict TF binding sites, infer regulatory network topologies, and define cell-type-specific expression programs from single-cell RNA-seq datasets [11]. These computational frameworks also contribute to the identification of predictive molecular signatures for disease diagnosis, prognosis, and therapy selection.

Moreover, recent advances in multi-omics integration, including single-cell epigenomics, spatial transcriptomics, and long-read sequencing, are enabling an unprecedented level of resolution of transcriptional regulation in health and disease. These technologies are unveiling spatiotemporal gene regulation patterns, context-specific enhancer activities, and cellular heterogeneity that were previously inaccessible with bulk approaches [12,13].

Taken together, these innovative strategies are reshaping the field of gene regulation and offer promising avenues for the development of targeted therapies aimed at correcting transcriptional dysregulation at its root.

This Special Issue highlights both the complexity and clinical relevance of transcriptional regulation, bringing together original research and reviews that span from hormone signaling and chromatin remodeling to ncRNAs and TF networks. Collectively, the contributions provide new mechanistic insights, reinforce the link between transcriptional misregulation and human diseases, and point toward emerging diagnostic and therapeutic strategies. We are confident that the findings presented here will stimulate further research and foster new interdisciplinary approaches aimed at understanding and correcting aberrant gene regulation in human pathology.

## Data Availability

Not applicable.
